# The regulatory network for petal anthocyanin pigmentation is shaped by the MYB5a/NEGAN transcription factor in *Mimulus*

**DOI:** 10.1093/genetics/iyaa036

**Published:** 2021-01-11

**Authors:** Xingyu Zheng, Kuenzang Om, Kimmy A Stanton, Daniel Thomas, Philip A Cheng, Allison Eggert, Emily Simmons, Yao-Wu Yuan, Gregory D Conradi Smith, Joshua R Puzey, Arielle M Cooley

**Affiliations:** 1 Departments of Biology and Applied Science, William & Mary, Williamsburg, VA 23185, USA; 2 School of Biological Sciences, Cold Spring Harbor Laboratory, Cold Spring Harbor, NY 11724, USA; 3 Department of Biology, Whitman College, Walla Walla, WA 99362, USA; 4 Department of Ecology and Evolutionary Biology, University of Connecticut, Storrs, CT 06269, USA

**Keywords:** anthocyanin, color patterning, differential expression, reaction-diffusion, Turing network, feedback loops, monkeyflower, Mendelian trait, evolution of novelty

## Abstract

Much of the visual diversity of angiosperms is due to the frequent evolution of novel pigmentation patterns in flowers. The gene network responsible for anthocyanin pigmentation, in particular, has become a model for investigating how genetic changes give rise to phenotypic innovation. In the monkeyflower genus *Mimulus*, an evolutionarily recent gain of petal lobe anthocyanin pigmentation in *M. luteus* var. *variegatus* was previously mapped to genomic region *pla2*. Here, we use sequence and expression analysis, followed by transgenic manipulation of gene expression, to identify *MYB5a—*orthologous to the *NEGAN* transcriptional activator from *M. lewisii—*as the gene responsible for the transition to anthocyanin-pigmented petals in *M. l. variegatus*. In other monkeyflower taxa, *MYB5a/NEGAN* is part of a reaction-diffusion network that produces semi-repeating spotting patterns, such as the array of spots in the nectar guides of both *M. lewisii* and *M. guttatus*. Its co-option for the evolution of an apparently non-patterned trait—the solid petal lobe pigmentation of *M. l. variegatus—*illustrates how reaction-diffusion can contribute to evolutionary novelty in non-obvious ways. Transcriptome sequencing of a *MYB5a* RNAi line of *M. l. variegatus* reveals that this genetically simple change, which we hypothesize to be a regulatory mutation in *cis* to *MYB5a*, has cascading effects on gene expression, not only on the enzyme-encoding genes traditionally thought of as the targets of *MYB5a* but also on all of its known partners in the anthocyanin regulatory network.

## Introduction

Anthocyanins, the red and purple pigments that color many plant tissues, are largely responsible for the tremendous visual diversity of floral pigmentation in angiosperms. The evolutionary lability of anthocyanin pigmentation, combined with its ecological and agricultural importance, has helped anthocyanin biosynthesis and regulation become a model for investigating the genetic mechanisms for trait evolution.

In dicots, the anthocyanin biosynthetic pathway is functionally divided into the early biosynthetic genes (EBGs) and the late biosynthetic genes (LBGs) (Dubos *et al.* 2010). The LBGs are coordinately activated by an MBW complex consisting of three kinds of proteins: a subgroup-6 R2R3 MYB protein (M) that directly binds the promoters of the enzyme-encoding genes, and bHLH (B) and WD40 (W) co-factors that physically interact with the MYB (Martin and Gerats 1993; Lepiniec *et al.* 2006; Petroni and Tonelli 2011; Xu *et al.* 2014; Zhu *et al.* 2015; Lloyd *et al.* 2017). An R3 MYB protein represses anthocyanin biosynthesis by sequestering the bHLH proteins and preventing them from taking part in MBW formation (Zhu *et al.* 2009; Yuan *et al.* 2013; Albert *et al.* 2014).

A growing body of empirical work has demonstrated that the anthocyanin regulatory network is highly interconnected. For example, the MBW complex triggers increased transcription of its own bHLH component, as well as increasing the expression of the R3 MYB repressor (Xu *et al.* 2015). In some cases, the MBW complex further upregulates the R2R3 MYB activator gene (Yuan *et al.* 2014). This positive feedback loop is essential to generating spatial patterns via a self-organizing series of molecular interactions described as a reaction-diffusion system.

In the 1950s, Alan Turing proposed such a reaction-diffusion model to explain the developmental origins of semi-regularly repeating patterns in biological organisms, such as the spots on a cheetah or the stripes on a zebra (Turing 1952), and the idea has been further developed by others (Gierer and Meinhardt 1972;  Meinhardt and Gierer 2000; Meinhardt 2012; Ball 2015). In contrast, traits that lack a repeating element are generally explained in terms of positional specification, in which overlapping zones of gene expression dictate the location of a physical feature (Nijhout 1991; Gompel *et al.* 2005; Werner *et al.* 2010; Martins *et al.* 2017).

Within the monkeyflower genus *Mimulus* (synonym *Erythranthe*; Barker *et al.* 2012; Lowry *et al.* 2019), floral anthocyanin traits have evolved repeatedly (Grossenbacher and Whittall 2011) and both types of patterning appear to be involved. The developmental genetics of floral anthocyanins have been studied in the greatest detail in *M. lewisii* and *M. guttatus*. In both of these species, the R2R3 MYB activator gene *NEGAN/MYB5* is part of a reaction-diffusion system that creates spots of red anthocyanin pigment in the nectar guide region (Ding *et al.* 2020). A paralagous R2R3 MYB gene, *PELAN*, similarly operates as part of an MBW complex in the petal lobe region of *M. lewisii*, with one important difference: *PELAN* lacks autocatalytic ability, and thus cannot initiate reaction-diffusion dynamics. Consistent with this finding, *PELAN* generates a continuous field of pigment within the positionally specified petal lobe region, rather than a repeated spotting pattern, and RNAi against *PELAN* causes a uniform lightening of pigmentation rather than the altered spatial pattern expected under a reaction-diffusion model (Yuan *et al.* 2014).

**Figure 1:**
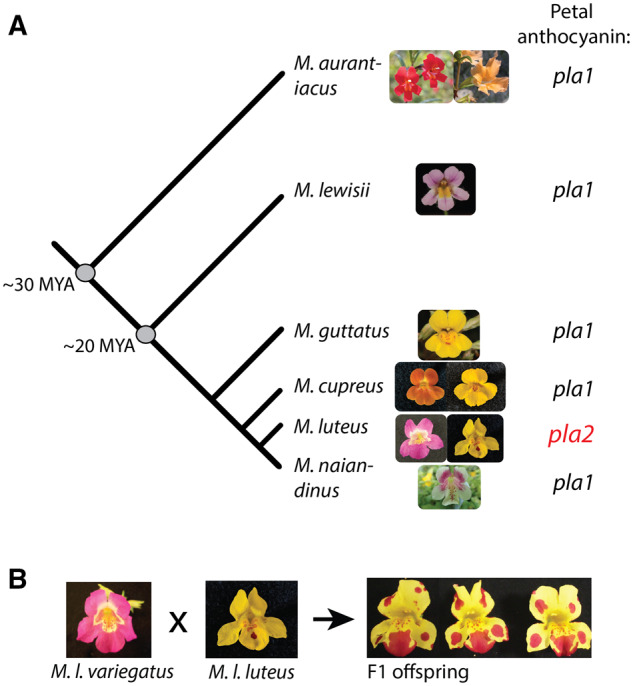
Petal anthocyanin pigmentation is evolutionarily labile within and between species of *Mimulus*. (A) Shifts in petal color in *Mimulus* have primarily been tracked to genomic region *pla1*. The causal gene in the *pla1* genomic region has been identified as the R2R3 MYB gene *MaMYB2* in *M. aurantiacus* (Streisfeld *et al.* 2013); its ortholog *PELAN* in *M. lewisii* (Yuan *et al.* 2014); and one or more R2R3 MYB genes at the orthologous genomic region in *M. guttatus* (Lowry *et al.* 2012), *M. cupreus* (Cooley *et al.* 2011), and *M. naiandinus* (A. Cooley, unpublished data). Genomic region *pla2* contains *MaMYB1* in *M. aurantiacus*; its ortholog *NEGAN* in *M. lewisii*; and *MYB5* in *M. guttatus*, *M. cupreus, M. l. variegatus*, and *M. naiandinus*. The two varieties of *M.luteus* are the purple-flowered *M. l.variegatus* (left) and the yellow-flowered *M. l. luteus* (right). Photo credits: *M. aurantiacus* courtesy of Matt Streisfeld; *M. naiandinus* courtesy of Melia Matthews. Divergence times not to scale. (B) Crosses between the two varieties of *M. luteus* reveal a spotting mechanism underlying the apparently solid purple pigmentation of *M. l. variegatus*, consistent with Turing-style reaction-diffusion.

At first glance, the solid purple pigmentation of *Mimulus luteus* var. *variegatus* (Figure 1A) would appear to be a classic case of positional specification. This evolutionarily recent, Mendelian trait shows no spotting, striping, or any other kind of the repetition that is thought to be the hallmark of reaction-diffusion traits. Surprisingly, when *M. l. variegatus* is crossed to its yellow-petaled conspecific, *M. l. luteus*, the offspring of the cross feature multiple spots in their petal lobes (Figure 1B). This altered spatial pattern suggests that the gain of petal lobe anthocyanin in *M. l. variegatus* is in fact a cryptic reaction-diffusion trait. And indeed, while solid petal color across the genus *Mimulus* maps to the genomic region containing the non-autocatalytic gene *PELAN* (*pla1* in Figure 1A), petal lobe pigmentation in *M. l. variegatus* maps to the region containing the reaction-diffusion activator gene *NEGAN* (*pla2* in Figure 1A).

In this work, we explore the gain of an apparently unpatterned trait via a reaction-diffusion activator gene that, in other *Mimulus* species, creates spotting patterns. We find that *MYB5a*, the ortholog of *NEGAN*, has been co-opted for petal lobe anthocyanin pigmentation while also retaining its ancestral function of activating anthocyanin spot patterns in the nectar guide. We report the first evidence of a positive correlation in expression between the R2R3 MYB activator and its WD40 co-factor, suggesting a possible new feedback loop within the MBW transcription-factor complex. Using transcriptome data in combination with mathematical modeling, we model the interaction between the R2R3 MYB activator, MYB5a, and the R3 MYB repressor, RTO.

The results highlight how a reaction-diffusion network, and its two resulting phenotypes of nectar guide and petal lobe pigmentation, can respond in a dramatic and coordinated fashion to a relatively modest change in the expression of a single component part. The estimates of interaction strengths will help to inform and constrain future mathematical models of the evolutionary and developmental systems that give rise to the color pattern diversity of flowering plants. Finally, we note that petal lobe anthocyanin in *M. l. variegatus*, which looks to all appearances like a traditional positionally specified feature, is in fact controlled by a reaction-diffusion network. The existence of such “cryptic” reaction-diffusion traits suggests that the importance of this mechanism to evolutionary and developmental biology may be substantially greater than previously supposed.

## Materials and methods

### Plant sources and growth conditions

Seeds were originally collected from natural populations in central Chile in 2004, and were inbred for multiple generations prior to the work described here (Table 1). Seeds were sown in 2-in pots onto wet Black Gold potting soil (SunGro Horticulture, Agawam, MA, USA) or Miracle-Gro potting soil (Scotts Miracle-Gro Co., Marysville, OH, USA), and were misted daily until germination. For transgenic experiments, seedlings were transplanted into 6″ pots to promote large size. Plants were maintained in the Whitman College greenhouse under 16-hour days and were watered daily by an automatic misting system. After expansion of the first true leaves, fertilizer (Grow Big, FoxFarm Soil & Fertilizer Co., Arcata, CA, USA, N:P:K = 6:4:4; Open Sesame, FoxFarm Soil & Fertilizer Co., Arcata, CA, USA, N:P:K = 5:45:19; and/or Miracle-Gro Bloom Booster, Scotts Miracle-Gro Co., Marysville, OH, USA, N:P:K = 1:3:2) was applied two to three times weekly to promote growth and flowering.

**Table 1:** Seed sources

Taxon	Line ID	Generations inbred	Population	Location
*M. luteus* var. *luteus*	Mll-EY7	12	El Yeso	33.4°S, 70.0°W (2600 m)
*M. luteus* var. *variegatus*	Mlv-RC6	11	Río Cipreses	34.2°S, 70.3°W (1200 m)

Locations from which seeds were collected are given as latitude, longitude (meters above sea level).

### Expression analyses of candidate anthocyanin-activating genes

**Figure 2:**
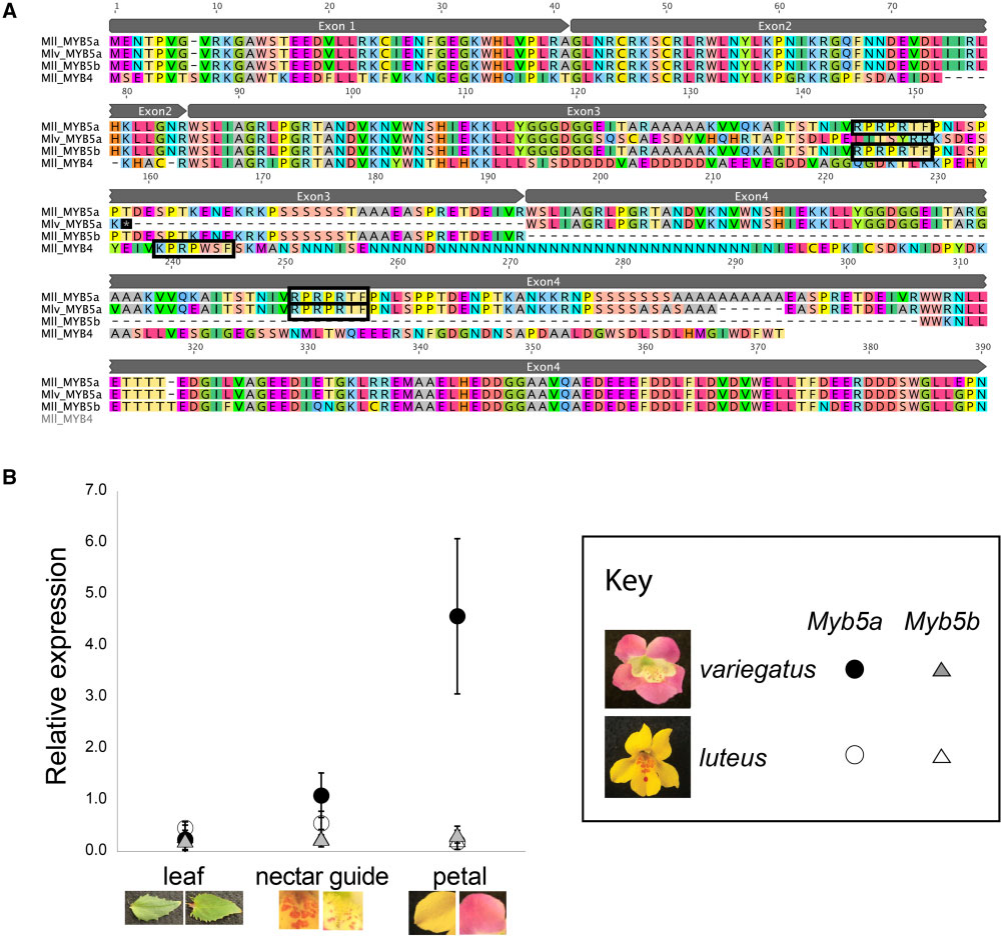
Sequence and expression of *MYB5a/NEGAN*. (A) Alignment of *MYB5a*, *MYB5b*, and *MYB4*. Amino acid sequences were inferred by translating DNA sequences, obtained from the *M. l. luteus* genome and transcriptome (Edger *et al.* 2017; Stanton *et al.* 2017) and from PCR and Sanger sequencing of *M. l. variegatus*. Black rectangles identify putative Subgroup 6 motifs. Note that this motif, which strongly predicts ability to activate anthocyanin production, is missing from *M. l. variegatus* exon 3. Gray boxes denote exon boundaries for MYB5a and MYB5b. Created in Geneious 9.1 by Biomatters (www.geneious.com). (B) qRT-PCR estimates of *MYB5a/NEGAN* and *MYB5b* expression in *M. l. variegatus* and *M. l. luteus*. Each point indicates the mean of three technical replicates, ±95% CI, relative to the reference gene *Actin* and a reference pool of cDNA.

Two candidate anthocyanin-activating genes, *MYB4* and *MYB5*, had been previously identified in *M. l. variegatus* by genetic mapping and sequence characterization (Cooley *et al.* 2011). Because members of the *luteus* complex are allotetraploids (Mukherjee and Vickery 1962; Vallejo-Marín *et al.* 2015; Edger *et al.* 2017), many genes have a homeologous copy located on a different chromosome, which could inflate expression estimates for a target gene if care is not taken to distinguish between the copies. In the process of investigating *MYB5* in the sequenced *M. l. luteus* genome, we discovered its apparent homeolog, *MYB5b* (Figure 2A). *MYB5* will therefore be referred to in this work as *MYB5a*, to distinguish it from its homeolog. In the *M. l. luteus* reference genome, the three exons that make up *MYB5b* are identical in their coding sequence to the first three exons of *MYB5a*. However, substantial divergence in the 5′-UTR (as well as downstream of the third exon) enabled the development of copy-specific primers, allowing us to disentangle the expression of *MYB5a* from its homeolog. We were not able to identify any homeolog of *MYB4* in the *M. l. luteus* genome.

The gene responsible for activating anthocyanin biosynthesis in *M. l. variegatus* petal lobes is expected to be expressed in developing floral buds, during or just prior to the accumulation of visible anthocyanin pigment. Developmentally, this occurs approximately 72 hours before anthesis (Cooley *et al.* 2011), before the bud has emerged from the calyx. Endpoint and quantitative RT-PCR were used to investigate patterns of expression for each candidate gene, as well as for *MYB5b*, in developing floral buds and also young leaves of the purple-lobed *M. l. variegatus* compared to the yellow-lobed *M. l. luteus*. Primers were designed based on the sequenced genome of *M. l. luteus* (Supplementary Table S1), and their functionality in *M. l. variegatus* was confirmed by PCR prior to their use in expression analyses.

Although *M. l. variegatus* and *M. l. luteus* have striking differences in petal lobe pigmentation, with abundant anthocyanin production in the former and none in the latter, they share the trait of anthocyanin spotting in the nectar guide region of the corolla. Petals of developing flower buds were therefore dissected into lobe *vs* nectar guide components and the two tissue types were analyzed separately.

RNA was extracted using the Agilent Plant RNA Isolation Kit (Santa Clara, CA, USA). cDNA was synthesized using the ProtoScript First Strand cDNA Synthesis Kit from New England BioLabs, Inc. (Ipswich, MA, USA). Endpoint (qualitative) RT-PCR was performed on cDNA, with gDNA template as a control for primer efficacy. In addition to the *MYB* primers, either *Actin* or *GAPDH* primers were included as a control for successful cDNA synthesis (Supplementary Table S1). PCRs were performed with 0.2 mM dNTPs, 0.2 μM of each primer, 0.2 μL of G-Biosciences Taq DNA Polymerase per 25-μL reaction, and 1× G-Biosciences buffer. Amplification was performed with temperature settings of 95°C for 3 min, followed by 30 cycles of (95°C for 30 s, variable temperature for 30 s, 72°C for 1 min per kb of product), and then 72°C for 10 min. The annealing temperature was set to 2°C lower than the lowest melting temperature of the set of primers used in the PCR, and the minimum extension time used was 30 s.

To test for expression patterns consistent with the activation of petal lobe anthocyanin synthesis, quantitative RT-PCR was performed on *MYB5a* and *MYB5b* using SYBR-Green Brilliant III Ultra-Fast reagents (Agilent Technologies, USA), in optical 96-well plates (Greiner Bio-One, Belgium), on an MxPro3000p analyzer (Stratagene, USA). Four biological replicates were used for each taxon and tissue type, collected from different plants of the same inbred line. Each biological replicate was run in triplicate and the average of the three technical replicates was used for statistical analyses. Samples were amplified for 40 cycles of 95°C for 10 s and 51°C for 20 s. Melt curves were obtained by heating from 51°C to 95°C with a ramp speed of 0.01°C per second. The *Actin* gene was used as a normalizer. Raw qPCR fluorescence data were collected and analyzed by the default settings of the MxPro software v.4.10 (Agilent Technologies, USA). Amplification efficiencies for each primer pair were determined using the Ct (threshold) values obtained from a 1/4 dilution series (1:4, 1:16, 1:64, 1:256, and 1:1024).

### Cloning and sequencing *MYB5a* splice variants

Two splice variants of *M. l. variegatus MYB5a*, containing exons 1-2-3 and 1-2-4 respectively, were PCR-amplified using primers MYB5a-10F and MYB5a-53R (Supplementary Table S1). PCR products were purified using a Genomic DNA Clean and Concentrate Kit (Zymo Research, Irvine, CA, USA), then cloned using the pGEM T-Easy kit and manufacturer’s protocol (Promega, Madison, WI, USA). pGEM vector containing the desired PCR product was transformed into competent JM109 *E. coli* cells (Promega, Madison, WI, USA). Colonies were evaluated using blue-white screening and PCR with M13 universal primers. Inserts of the expected length were sent to Eton Bioscience (San Diego, CA, USA) for Sanger sequencing.

### Transgene construction

A previously built overexpression construct (Yuan *et al.* 2014), containing the complete *NEGAN* coding sequence from *M. lewisii*, driven by a 35S promoter, was used to test the sufficiency of *MYB5a* for activating petal lobe anthocyanin biosynthesis in *M. l. luteus*, a variety that normally lacks petal lobe anthocyanin pigmentation (Figure 1A).

Two RNAi constructs were built, targeting *M. l. variegatus MYB5a*-specific sequences in the 5′-UTR and fourth exon respectively, using the Gateway method (Earley *et al.* 2006; Katzen 2007). In brief, each fragment of interest was PCR-amplified using a forward primer with a 5′-CACC tag. The tag permitted the fragment to be directionally cloned into a pENTR entry vector (Thermo Fisher Scientific, Waltham, MA, USA); attL/attR recombination was used to transfer the correctly oriented fragment into the Gateway-compatible destination vector pB7GWIWG2(I) (VIB, Ghent, Belgium). *M. l. variegatus* genomic DNA for PCR was extracted using the E.Z.N.A. SP Plant DNA Kit (Omega Bio-Tek, Norcross, GA, USA). PCR amplification was performed using Phusion High Fidelity DNA Polymerase (New England Biolabs). The inserts and primers used for RNAi transgene construction are summarized in Table 2.

**Table 2:** Construction of *MYB5a/NEGAN* RNAi transgenes for use in *M. l. variegatus*

Insert	Insert length (bp)	Primers
*M. l. variegatus* 5′-UTR fragment	307	24 F: caccCAAATTTTGGTTTTTGCCATTT
		37 R: TCGGTCAAATTAAATGCACA
*M. l. variegatus* exon 4 fragment	377	60 F: caccGAAGAACGGCCAACGAC
		56 R: GCAGCTTCCCCGTTTCA

Both inserts were cloned into the pB7GWIWG2(I) RNAi vector. Primers are labeled with a number and F for Forward or R for Reverse. Their sequences are shown 5′ to 3′. Nucleotides added to primers for purposes of directional cloning or restriction enzyme digests are underlined. bp, base pairs. Lowercase cacc- represents nucleotides added to primers to enable cloning into the pENTR entry vector.

### Stable transformation

Agrobacterium-mediated plant transformation was performed using floral spray and vacuum infiltration as described in Yuan *et al.* (2013). Each experiment utilized approximately 12 robustly budding 2- to 6-month-old plants growing in 6″ pots. The commercial *Agrobacterium tumefaciens* strain LBA4404 (ThermoFisher Scientific) produced only two transformed seedlings from the infiltration of 67 plants (K. Om, senior thesis 2017). Subsequent experiments were performed using strain GV3101, and this yielded higher rates of transformed seedlings.

The concentration of Silwet-L77 (Lehle Seeds, Round Rock, TX, USA) used by Yuan *et al.* (2013) was 0.1%, or 1 mL per L of *Agrobacterium* culture. This unusually high concentration of Silwet was previously found to increase transformation success in *Mimulus* (Y.-W. Yuan, unpublished data). Acetosyringone was added to a final concentration of 0.1 M as in Yuan *et al.* (2013).

The predominantly outcrossing taxa *M. l. luteus* and *M. l. variegatus* were manually self-pollinated for 2 weeks after the first post-infiltration flower opened. Seeds were collected and densely sown in 25 cm × 50 cm flats. As soon as germination was observed, flats were sprayed daily with 1:1000 Finale (Farnam Companies, Inc., Phoenix, AZ, USA) to eliminate non-transgenic plants. Transgenic seedlings were grown to flowering and photographed. gDNA was extracted and the presence of the transgene was verified by PCR, using a forward primer targeted to the transgene and a reverse primer targeted to the attR2 region of the transgenic plasmid (Table 2 and Supplementary Table S1).

RNA extractions, cDNA synthesis, and endpoint RT-PCR were performed as described in the Expression Analyses section, to evaluate the expression of the target gene relative to an untransformed control plant. Two different primer pairs targeting *MYB5a* were used, 33 F-40R and 44 F-45R, along with primers to reference gene *GAPDH* (Stanton *et al.* 2017) as a positive control (Supplementary Table S1).

### RNA extraction and library preparation for RNAseq

Transcriptome analysis was used to confirm the specific knockdown of target gene *MYB5a* by RNAi, and to explore the effects of this knockdown on other anthocyanin-related genes. Petal tissue for transcriptome sequencing was collected separately from three individuals of our highly inbred wild-type line of *M. l. variegatus*, RC6, and from three white-flowered offspring (Vrnai1.1, 1.3, and 1.5; Supplementary Figure S2) of the white-flowered RNAi transformant Vrnai1. For comparative purposes, one sample from *M. naiandinus* (Figure 1A) developing petals was also included.

Because the expression of anthocyanin-producing genes in *Mimulus* flowers is highest early in bud development, just before and after the first appearance of visible anthocyanin pigment (Cooley *et al.* 2011; Yuan *et al.* 2014), we used young buds that had not yet emerged from the calyx. Anthers were removed and the remaining petal tissue (including both lobe and nectar guide regions) was snap frozen in liquid nitrogen. RNA was extracted from each of the six samples using the Agilent Plant RNA Isolation Kit (Wilmington, DE, USA). A stranded RNA-Seq plus Ribo-Zero library preparation, followed by one lane of Illumina HiSeq 4000 50-bp single-read sequencing, was performed by the Duke University sequencing core facility (Durham, NC, USA). The FASTQ format RNA libraries were deposited in the NCBI Sequence Read Archive (SRA) repository under accession number PRJNA629107.

### Transcriptome alignment

With a published *M. l*. *luteus* genome and gene feature annotation file available (Edger *et al.* 2017), we chose the genome splice-unaware mapping approach to assemble the *M. l*. *variegatus* transcriptomes to the *M. l. luteus* genome (Stein 2011; Williams *et al.* 2014). Sequencing quality control was performed by plotting the sequence nucleotide distribution and sequencing quality scores for all samples. Transcriptome libraries were aligned using Bowtie2(Version 2.3.5.1) under -very-sensitive-local mode (Langmead and Salzberg 2012) for best results in distinguishing homeolog expression, given that the *M. luteus*-group species are putative tetraploids (Mukherjee and Vickery 1962; Vickery 1995). Overall alignment rates were greater than 90% for all 7 samples. From the sequence alignment maps we counted reads per gene for all samples using the exon coordinates included in the published luteus GFF (general feature format) file. Read count was performed with software HT-seq (Version 0.11.2) (Anders *et al.* 2014) in Python. For detailed alignment documents, see the Supplementary Data. Pipeline code, alignment documents, and results are accessible through the GitHub repository (https://github.com/cici-xingyu-zheng/Luteus-RNA-seq) and for raw read count, see Supplementary Table S2.

### Transcriptome analysis and functional annotation

Analyses of differential gene expression were conducted in DESeq2 (Version 1.26.0) in the R/Bioconductor environment (Love *et al.* 2014) (R Version 3.6.2; Bioconductor Release 3.10). After normalizing each gene by sequencing depth, we performed a principal component analysis plot of the seven transcriptome samples (three wild-type *M. l. variegatus*, three *M. l. variegatus* from RNAi line Vrnai1, and *M. naiandinus* as an outgroup). As expected, the samples clustered by treatment with the outgroup being an outlier (Supplementary Figure S2).

After performing a shrinkage estimation for dispersion to address the inaccuracy introduced by the small sample size and reduce the false positive rate (Love *et al.* 2014), the logarithmic fold change between RNAi treatment and control samples was used to evaluate differential expression. Following a false discovery rate control using the Benjamini–Hochberg Correction method, transcripts that were log-2-fold up- or down-regulated with a *P-*value <0.05 were considered to be significantly differentially expressed. Transcript expression profiles were normalized to reads per kilobase per million (RPKM) for further analysis and for plotting (Supplementary Table S3).

To annotate differentially expressed genes, all gene sequences from the *M. l. luteus* reference genome were translated to protein sequences using EMBOSS(6.5.7) “transeq” command and searched against *Arabidopsis thaliana* (TAIR) protein database (www.arabidopsis.org) using the “BLASTp” query with a e-value cut-off <10^−6^. For each coding sequence from *M. l. luteus*, the best-hit *A. thaliana* gene was used to annotate the transcript with a gene name and Gene Ontology (GO) annotation terms.

These annotations were then applied to the *M. l. variegatus* transcriptome data. GO enrichment analysis for was conducted, for genes that were differentially expressed between wild-type *M. l. variegatus* and the *MYB5a* RNAi line Vrnai1, using topGO (Version 2.36.0) with the “org. At.tair.db” database in R. Pathway enrichment tests were done using the KEGG (the Kyoto Encyclopedia of Genes and Genomes, https://www.genome.jp/kegg/;Release 93.0) pathway assignments for Arabadopsis with the KEGGREST Bioconductor package (Kanehisa* et al*.,^ 2016^).

### Tree construction

A gene tree was constructed to determine the relationship of Mlu_12200, the candidate *MYB5a* gene, to previously studied *Mimulus* MYBs. Sequences used to construct the gene tree in Figure 2 of Yuan *et al.* (2014) were downloaded from GenBank. A full list of GenBank sequence IDs are available in the Supplementary Table S4. Amino acid sequences were aligned with Clustal Omega and an ML gene tree was constructed using PhyML using a WAG substitution model and 100 bootstrap replicates. After initial tree construction, a subset of sequences that formed the core clade of MYBs of interest was realigned using Clustal Omega. The alignment cleaned manually to remove poorly aligned regions, and a tree was constructed using PhyML. The full gene tree from this analysis is available in the Supplementary Figure S3.

### Regulatory and pathway analysis

In order to test for an effect of *MYB5a* down-regulation on the anthocyanin biosynthetic pathway, we examined the expression of genes corresponding to six core pathway enzymes that produce the cyanidin pigment found in *M. l. variegatus* (Cooley *et al.* 2008). These are: chalcone synthase (CHS), chalcone isomerase (CHI), flavonoid-3-hydroxylase (F3H), dihydroflavonol-4-reductase (DFR), anthocyanidin synthase (ANS), and UDP-flavonoid-3-glucosyl-transferase (UF3GT) (Holton and Cornish 1995; Tanaka *et al.* 2008). A list of *M. l. variegatus* transcripts with *RPKM* >1 and annotated with descriptions matching these enzymatic activities was obtained. Genes were considered truly orthogonal if they were the best hit in a reciprocal BLAST or had the same high BLAST score as a putative homeolog.

In addition to the enzyme-encoding genes, we were also interested in what other regulatory factors might be affected when we knock down the *MYB5a* transcriptional activator. In all species that have been examined, the regulatory complexes of the anthocyanin pathway include members of the MYB, basic helix-loop-helix (bHLH), and WD40 repeat families (Dooner *et al.* 1991; Koes *et al.* 2005). Therefore, we subset from the differentially expressed genes a list of genes annotated as members of these three transcriptional regulator families based on comparison to *A. thaliana*. Within this subset, we used reciprocal best 267 BLAST hits to identify orthologs to the genes that reported to be involved in 268 anthocyanin regulation in *M. guttatus* and *M. lewisii*.

### Data availability

Seeds, bacterial strains, and plasmids are available upon request. Individual *MYB4, MYB5a*, and *MYB5b* coding sequences are located in GenBank. Whole-genome sequence for *M. l. luteus* was released by Edger *et al.* (2017) along with the draft genome and are located at https://datadryad.org/stash/dataset/doi:10.5061/dryad.d4vr0. Raw transcriptome data for *M. l. luteus* are published in Stanton *et al.* (2017) and are located at http://dx.doi.org/10.5061/dryad.84655. The *M. l. variegatus* transcriptome raw reads are available on the Sequence Read Archive (https://www.ncbi.nlm.nih.gov/bioproject/PRJNA629107/). Transcriptome analysis code and alignment documents are accessible through the GitHub repository (https://github.com/cici-xingyu-zheng/Luteus-RNA-seq).

Supplementary material is available at figshare DOI: https://doi.org/10.25386/genetics.13350800.

## Results

### Expression of an alternately spliced R2R3 MYB, *MYB5a/NEGAN*, covaries with petal anthocyanin

In *M. l. variegatus*, only two anthocyanin-related genes were found within the genetically mapped petal anthocyanin locus *pla2* (Cooley *et al.* 2011). The two genes, *MYB4* and *MYB5a*, show phylogenetic similarity to the anthocyanin-activating Subgroup 6 of the R2R3 MYB gene family (Stracke *et al.* 2001; Cooley *et al.* 2011). Reciprocal BLAST searches of the *M. lewisii* genome indicate that *MYB5a* is orthologous to *NEGAN*, a gene shown to be responsible for nectar guide anthocyanin spots in *M. lewisii* (Yuan *et al.* 2014). *NEGAN* has previously been shown to be orthologous to *MYB5a* from *M. guttatus* (Yuan *et al.* 2014).

*MYB4*, while somewhat similar in sequence to *MYB5a/NEGAN*, has a nonconservative amino acid substitution in the functionally critical DNA-binding motif that is a hallmark of Subgroup 6 transcription factors (Figure 2A) and has a top BLAST hit on NCBI to *M. guttatus* LOC105953416, which encodes a GL1-like trichome differentiation protein. Additionally, unsuccessful efforts to amplify *MYB4* out of *M. l. variegatus* floral bud cDNA, using primers that successfully amplify the gene from gDNA, indicate that this gene is not detectably expressed in developing *M. l. variegatus* buds (Supplementary Figure S1). Consistent with its similarity to a trichome differentiation gene, we did detect expression of *MYB4* in leaf tissue (Supplementary Figure S1).

In contrast, quantitative RT-PCR revealed that *MYB5a/NEGAN* is strongly and specifically expressed in the anthocyanin-pigmented petal lobes of *M. l. variegatus*, while showing little to no expression in the non-anthocyanin-pigmented petal lobes of *M. l. luteus* (Figure 2B). Expression of *MYB5a/NEGAN* was modest in the anthocyanin-pigmented nectar guide tissue of both taxa, and undetectable in their leaf tissue (Figure 2B). The homeologous gene copy, *MYB5b*, showed consistently low expression across both taxa and all tissue types, at levels less than 10% of that observed in *M. l. variegatus* petal lobes (Figure 2B).

The anthocyanin-activating subgroup 6 R2R3 MYBs generally have three exons. The diagnostic Subgroup 6 amino acid motif is KPRPR[S/T]F in *Arabidopsis*, and it is located in the third exon (Stracke *et al.* 2001). In *M. l. luteus* and *M. l. variegatus*, however, *MYB5a* genomic sequence data revealed the presence of a fourth exon that is extremely similar in sequence to the third exon. In both taxa, this fourth exon appears to be intact and potentially functional, including the Subgroup 6 motif. The fourth exon differs between *M. l. luteus* and *M. l. variegatus* by 7 amino acid substitutions and one in-frame indel (Figure 2A). The third exon, although apparently intact in *M. l. luteus*, has a 26-bp frameshift-inducing deletion in *M. l. variegatus* that eliminates the critical Subgroup 6 domain, alters 35 amino acids, and creates a premature stop codon (Figure 2A). Exons one and two are identical between the taxa.

RT-PCR, followed by cloning and Sanger sequencing, revealed that exons 3 and 4 represent two alternate splice variants of *MYB5a*. An “exon 1-2-3” transcript was occasionally isolated from developing *M. l. variegatus* petals, but was never found in *M. l. luteus*, even in the anthocyanin-pigmented nectar guides. An “exon 1-2-4” transcript was reliably isolated from *M. l. variegatus* petal lobes and from the spotted nectar guide tissue of the corolla throat. In *M. l. luteus*, the exon 1-2-4 transcript was recovered from nectar guides but not from the (anthocyanin-free) petal lobe tissue. Thus, the exon 1-2-4 transcript of *MYB5a* appears to be consistently expressed in all anthocyanin-pigmented floral tissue, and absent from tissue that lacks anthocyanin.

### *MYB5a/NEGAN* is sufficient and necessary for activating petal anthocyanin in *M. luteus*

**Figure 3:**
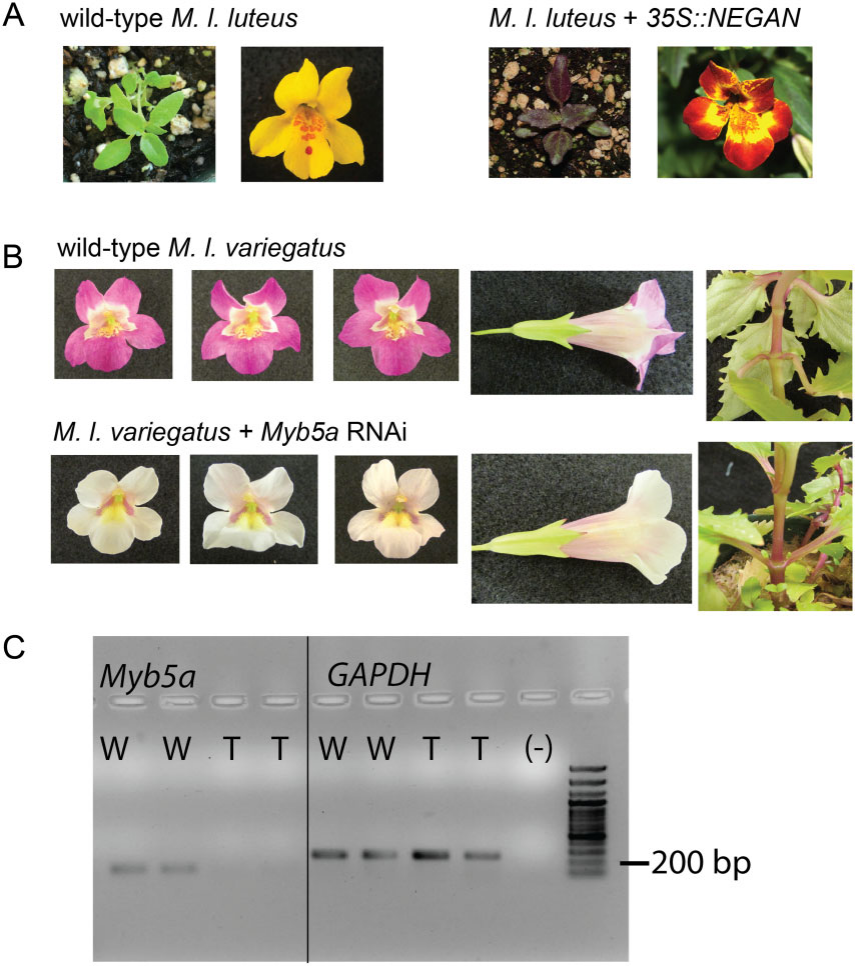
*MYB5a/NEGAN* is sufficient and necessary for activation of petal lobe anthocyanins. (A) Overexpression of the coding sequence of *MYB5a/NEGAN* from *M. lewisii* in the normally yellow-petaled *M. l. luteus* activates anthocyanin biosynthesis in both leaf and petal tissue. (B) RNAi targeting *MYB5a* exon 4 in the normally purple-petaled *M. l. variegatus* eliminates anthocyanin biosynthesis in the petal lobes and nectar guides, but not elsewhere in the plant. The strongest of 8 RNAi lines, Vrnai1, is shown here. See Supplementary Data for images of the other RNAi lines. (C) Qualitative (end-point) RT-PCR on cDNA from developing petal lobes of wild type (W) and transgenic (T) *M. l. variegatus* reveals a reduction in *MYB5a* expression in the Vrnai1 transgenic line. Reference gene *GAPDH* was used as a positive control.

A pilot experiment showed that *NEGAN* from *M. lewisii*, when overexpressed with the 35S promoter, was sufficient to activate anthocyanin production in the petal lobe, as well as the nectar guide region and leaf tissue, of the normally yellow-petaled *M. l*. *luteus* (Figure 3A). Encouraged by this result, we used a 35S-driven RNAi transgene to test the hypothesis that the exon 1-2-4 transcript of *MYB5a* is necessary for petal lobe anthocyanin pigmentation in the purple-petaled *M. l. variegatus*. In the strongest RNAi line, “Vrnai1,” anthocyanin pigmentation was completely abolished in both the petal lobe and the nectar guide (Figure 3B). Anthocyanin pigmentation in the stems, the leaves, and the adaxial side of the corolla appeared to be unaffected, indicating that the effect of *MYB5a* knockdown is spatially specific. Successful knockdown of *MYB5a* in line Vrnai1 was demonstrated via qualitative RT-PCR (Figure 3C), and confirmed through transcriptome analysis (next section).

**Figure 4:**
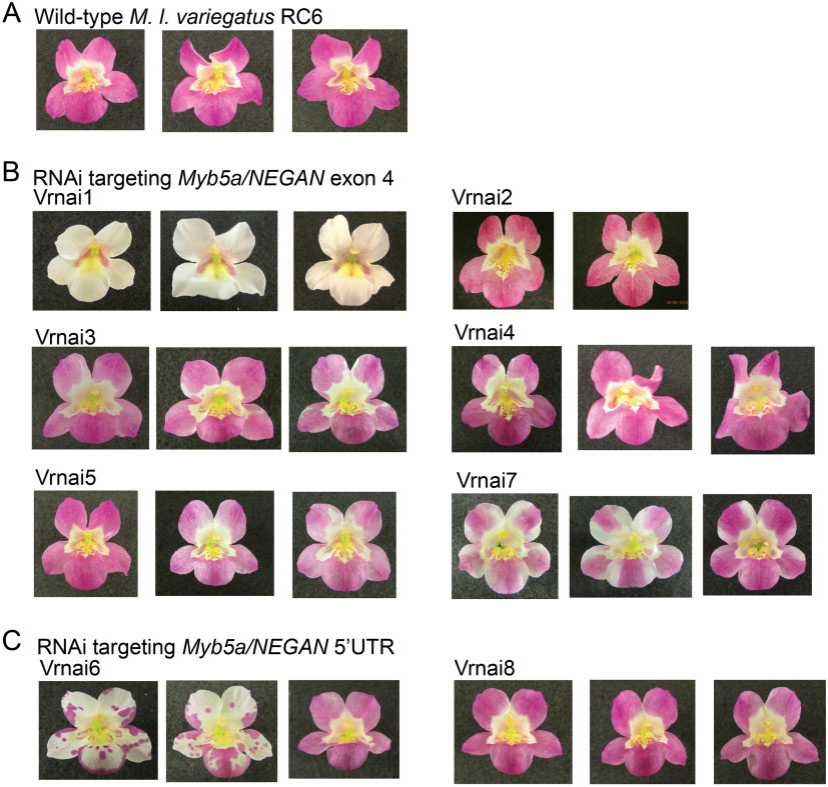
Transgene effects vary between and within insertion lines. (A) Wild-type *M. l. variegatus*. (B) Stable transgenic lines, for which the presence of an RNAi transgene targeting *MYB5a/NEGAN* exon 4 was confirmed by genotyping. (C) Stable transgenic lines, for which the presence of an RNAi transgene targeting *MYB5a/NEGAN* 5′-UTR was confirmed by genotyping.

Considerable variation in the severity of the RNAi phenotype was observed across different transgenic lines, as is typical for this technique (Figure 4). From 12 wild-type *M. l. variegatus* transformed with RNAi targeting exon 4 of *MYB5a*, six putatively transgenic seedlings were obtained. Four of these (Vrnai1, Vrnai3, Vrnai5, Vrnai7) had a visible reduction in petal lobe anthocyanin pigmentation. From an additional 12 wild-type *M. l. variegatus* transformed with RNAi targeting the 5′-UTR of *MYB5a*, two putatively transgenic seedlings were obtained. One of these (Vrnai6) had a visible reduction in petal lobe anthocyanin pigmentation. Genotyping with a *MYB5a* forward primer and the attR2 transgene-specific reverse primer confirmed the presence of the RNAi transgene in all eight lines. In line Vrnai6, the RNAi phenotype declined over the lifetime of the plant (Figure 4). This phenomenon has been shown to be caused by methylation and epigenetic silencing, particularly of the 35S promoter, in both *A. thaliana* and the tobacco species *Nicotiana attenuata* (Daxinger *et al.* 2008; Mlotshwa *et al.* 2010; Weinhold *et al.* 2013).

The Vrnai1 plant was self-fertilized, and eight of the resulting seeds were planted and grown to flowering. As expected for a single-copy (heterozygous) insertion of a dominantly acting transgene in the Vrnai1 plant, we observed a 3:1 ratio of six white-flowered and two wild-type plants among the offspring (Supplementary Figure S2). The six white-flowered offspring exhibited slight anthocyanin mottling on some flowers (Supplementary Figure S2).

### Transcriptomic differences between wild type and *MYB5a* RNAi lines of *M. l. variegatus*

We found a total of 632 genes that were significantly differentially expressed between wild-type and Vrnai1 lines of *M. l. variegatus*, with 346 genes down-regulated and 290 up-regulated in the Vrnai1 line (Supplementary Table S5). The differentially expressed genes are enriched in a variety of functions including response to UV-B, anthocyanin-containing compound biosynthesis, pollen exine formation, and phenylpropanoid biosynthetic process (*P*-value <0.001) (Supplementary Table S6). A pathway enrichment analysis identified two significantly enriched pathways (Supplementary Table S7): 1. cutin, suberine and wax biosynthesis, and 2. flavonoid biosynthesis, which includes the anthocyanin biosynthetic pathway (Holton and Cornish 1995).

**Figure 5:**
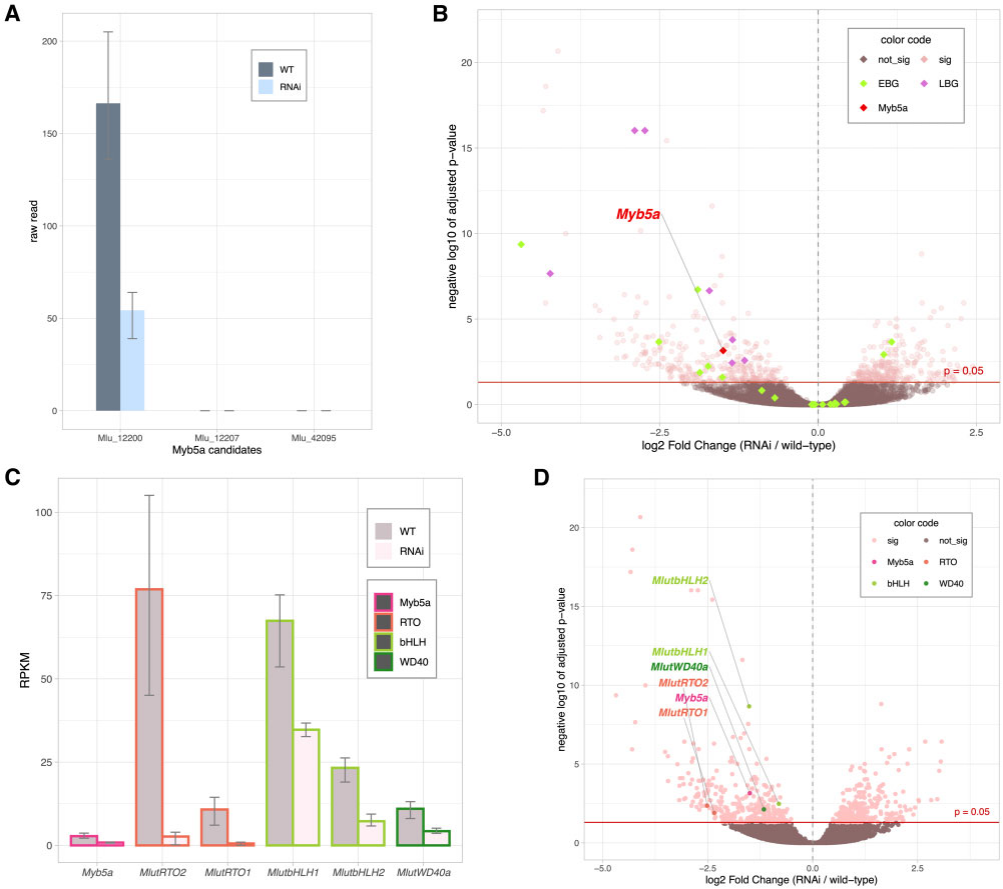
RNAi knockdown of *MYB5a* (transcript Mlu 12200) coordinately down-regulates Late Biosynthetic Genes and the entire anthocyanin regulatory network. (A) Transcript Mlu 12200 corresponds to *Myb5a/NEGAN*. Bars show average read-count (*n* = 3) of wild-type and RNAi lines respectively, for candidate transcripts Mlu 12200, Mlu 12207, and Mlu 42095. Error bars mark the highest and lowest read counts; only Mlu 12200 has any mapped reads. (B) Differentially expressed anthocyanin biosynthesis genes. Green, EBGs (Early Biosynthetic Genes). Purple, LBGs (Late Biosynthetic Genes). Log-2-fold change (RNAi *vs* wild-type) is shown on the *x* axis, and −log 10 of adjusted *P*-value on the *y* axis. (C) Transcript level fold change of anthocyanin regulators. Normalized expression fold change is shown in RPKM (per million mapped reads). Error bars represent maximum and minimum expression levels among the samples (*n* = 3). (D) The six regulatory genes of interest (*Myb5a*, *MlutbHLH1*, *MlutbHLH2*, *MlutRTO1*, *MlutRTO2*, and *MlutWD40a*) are highlighted in the gene expression volcano plot.

**Figure 6:**
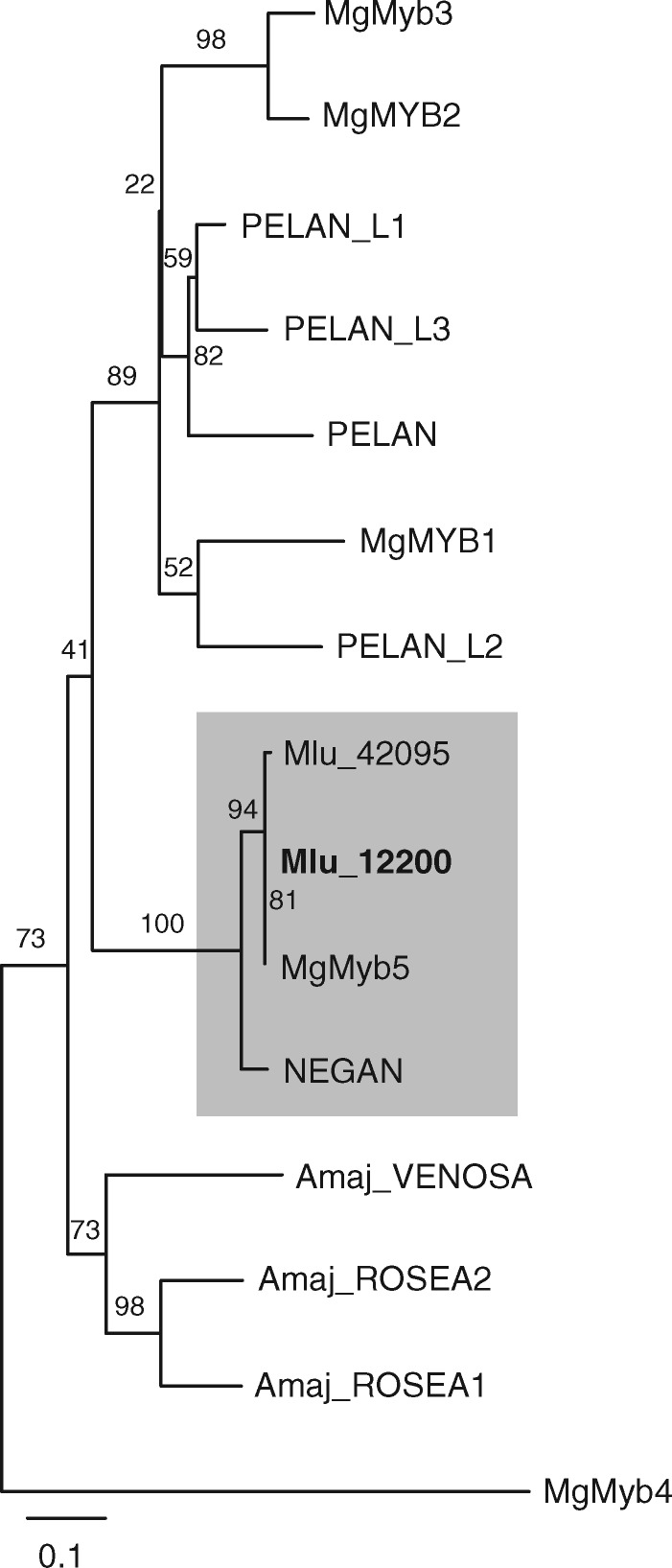
Maximum likelihood tree of anthocyanin-related R2R3 MYBs. Mg, *Mimulus guttatus*; Mlu, *Mimulus luteus*; Amaj, *Antirrhinum majus*. Mlu_12200 was identified as the anthocyanin activator MYB5a on the basis of its grouping with *M. guttatus MYB5* and *M. lewisii NEGAN*, and its patterns of expression in developing petal tissue from wild-type *vs MYB5a* RNAi lines of *M. luteus* var. variegatus. Its paralog, Mlu_42095, is not detectably expressed in the developing anthocyanin-pigmented petals of *M. l. variegatus*. Scale bar indicates substitutions per site. See Supplementary Figure S3 for an untrimmed tree containing additional taxa and genes.

The *M. l. variegatus MYB5a* DNA sequence, which had previously been determined by PCR and Sanger sequencing, had best hits to three coding sequences in the *M. l. luteus* reference genome: Mlu 12200, Mlu 12207, and Mlu 42095. In the *M. l. variegatus* transcriptomes, the latter two transcripts were not detectably expressed in developing petal tissue, in either wild-type or RNAi lines of *M. l. variegatus*, eliminating them from further consideration as the petal anthocyanin activator. In contrast, Mlu 12200 showed expression patterns consistent with our expectation for *MYB5a*: it was robustly expressed in all six libraries, with threefold higher expression in the wild-type compared to Vrnai1 (Figure 5A). Both the Mlu 12200 and the Mlu 42095 transcripts from *M. l. variegatus* grouped phylogenetically with *M. guttatus MYB5* and *M. lewisii NEGAN* (Figure 6).

To check for off-target effects of the RNAi transgene, we asked whether any other Subgroup 6 R2R3 MYB genes were significantly down-regulated in the RNAi line. Five MYBs were identified among the down-regulated genes (Mlu 24690, Mlu 05348, Mlu 27563, Mlu 17841, and Mlu 00921), but none of them contained a Subgroup 6 motif. We conclude that the loss-of-pigment phenotype observed in line Vrnai1 is due to the reduction in *MYB5a* expression alone, and was not caused by incidental down-regulation of another anthocyanin-activating MYB gene.

### RNAi knockdown of *MYB5a* reduces transcription of the late anthocyanin biosynthesis genes

We identified a total of 30 genes that are annotated to have enzymatic functions corresponding to the six core enzymes in the cyanidin pathway (Holton and Cornish 1995) (Supplementary Table S8). The EBGs (*CHS*, *CHI*, and *F3H*) are particularly enriched in copy number, each having more than the two homeologous copies expected in a tetraploid.

In eudicots, the anthocyanin biosynthetic genes are usually divided into two groups, the early (EBGs) and late (LBGs) biosynthetic genes, with the latter being tightly regulated by an MBW complex (Martin and Gerats 1993; Lepiniec *et al.* 2006; Dubos *et al.* 2010; Petroni and Tonelli 2011; Xu *et al.* 2014; Lloyd *et al.* 2017). In Arabidopsis, *CHS*, *CHI*, and *F3H* belong to the EBGs, producing the precursors of not only flavonoid pigments but other flavonol compounds. *DFR*, *ANS*, and *UF3GT* comprise the LBGs. Conserved as the enzymatic pathway is, though, the break point of regulation between early and late genes can vary across species (Streisfeld and Rausher 2009). We utilized our transcriptomic data to determine where the breakpoint occurs in *M. l. variegatus*.

Consistent with expectations from the literature, the anthocyanin biosynthetic genes in *M. l. variegatus* show a dichotomous pattern of response to *MYB5a* down-regulation. The genes earlier in the pathway—*CHS*, *CHI*, and *F3H—*had no consistent pattern of expression change (Figure 5B and Supplementary Figure S4), while the genes later in the pathway—*DFR*, *ANS*, and *UF3GT—*were consistently down-regulated in Vrnai1 (Figure 5B and Supplementary Figure S5). All copies of the LBGs had statistically significant change in the same direction (lower expression in the *MYB5a* RNAi line relative to wild-type), while the multiple copies of the EBGs showed a mix of expression increase, decrease, and no change in the RNAi line. This breakpoint is consistent with observations in the congeneric *M. aurantiacus* (Streisfeld *et al.* 2013). In summary, the results suggest that *MYB5a* controls anthocyanin production by regulating the LBGs, which include multiple copies of *DFR*, *ANS*, and *UF3GT*.

### RNAi knockdown of *MYB5a* reduces transcription of other anthocyanin regulators

Within the list of differentially expressed genes, we identified 20 genes that are annotated to be transcription factors with MYB or helix-loop-helix (HLH) domains, or that encode a WD40 protein that could potentially belong to a MYB–bHLH–WD40 regulatory complex (Supplementary Table S9). Since different MBW genes regulate a variety of traits besides anthocyanin synthesis and gene functions might have diverged between *Mimulus* and *Arabidopsis*, we further compared this subset of genes to the MBW regulatory genes within *M. lewisii*, the *Mimulus* species with the most thoroughly characterized anthocyanin regulatory network. One WD40 gene (labeled *MlutWD40a*) was the best hit to *M. lewisii* MlWD40a, a gene that co-activates anthocyanin expression in the corolla. Two bHLH genes (*MlutbHLH1* and *MlutbHLH2*) have the highest hit to the same bHLH gene in *M. lewisii*, *ANbHLH3*, indicating they are duplicates of each other. Interestingly, in *M. lewisii*, *ANbHLH3* is not detectably expressed in the petal lobes, suggesting a functional diversification in the bHLH co-factors as well as in *MYB5a/NEGAN* (Yuan *et al.* 2014).

Recent studies show that single-repeat R3 MYBs are activated by the R2R3 MYB member of the MBW complex, and inhibit anthocyanin biosynthesis by directly interacting with the bHLH component of the same complex. In two *Mimulus* species, *M. lewisii* and *M. guttus*, the same R3 MYB (RTO) is reported to down-regulate floral anthocyanin production. We found that two R3 MYB genes, Mlu 13044 (*MlutR3MYB1*) and Mlu 33990 (*MlutR3MYB2*), both have the highest BLAST hit to this RTO gene and are strongly and significantly down-regulated in the *MYB5a* RNAi line, Vrnai1. Their down-regulation in Vrnai1 is consistent with the model that the R2R3 MYB, *MYB5a*, is an activator of its own inhibitor, although the impact of *MYB5a* on two apparently homeologous gene copies is unique.

The activating properties of the anthocyanin-activating R2R3 MYB on itself, its R3 MYB inhibitor, and its bHLH cofactor, have been previously shown in other taxa (Yuan *et al.* 2014; Xu *et al.* 2015). We hypothesized that in *M. l. variegatus* RNAi lines, the bHLH and RTO candidate anthocyanin regulators that we identified would show the same directional expression change as *MYB5a* (Li 2014). In fact, our expression results showed that down-regulating *MYB5a* in the RNAi line of *M. l. variegatus* led to a down-regulation in all the putative regulatory genes (Supplementary Table S10), surprisingly including the WD40 genes (Figure 5, C and D). The involvement of the WD40 genes in a positive feedback loop has not, to our knowledge, been reported previously. The activators of anthocyanin biosynthesis—including R2R3 MYB, bHLH, and WD40 transcription factors—were all two- to threefold down-regulated in the RNAi line. The two R3 MYB inhibitors showed much more dramatic effects: they were 19- and 29-fold down-regulated (Figure 5, C and D).

## Discussion

Here we demonstrate that *MYB5a* is both sufficient and necessary for the evolutionarily recent, single-locus gain of petal lobe anthocyanin within *M. luteus*. We identify an unusual four-exon structure to this Subgroup 6 R2R3 MYB, along with evidence for alternative splicing. While the role of coding *vs cis*-regulatory evolution (Hoekstra and Coyne 2007; Stern and Orgogozo 2008) was not explicitly examined in this paper, we found that patterns of expression of *MYB5a* corresponded to pigmentation: the gene was strongly expressed in the heavily anthocyanin-pigmented petal lobes of *M. l*. *variegatus*; not detectably expressed in the petal lobes of *M. l. luteus*, a conspecific which lacks petal lobe anthocyanin; and modestly expressed in the partly pigmented nectar guides of both taxa. Further supporting the importance of expression change rather than coding change is that the orthologous coding sequence from the evolutionarily distant *M. lewisii*, which is approximately 20 MY divergent from *M. l. variegatus* (Nie *et al.* 2006), was highly effective at activating anthocyanin in the normally yellow-flowered *M. l. luteus*. We therefore predict that the causal mutation is either *cis* to *MYB5a*, or located in a *trans*-regulator of *MYB5a* that is closely linked to *MYB5a* within the mapped *pla2* genomic region. Additional transgenic experiments, such as coding or promoter swaps, will help to clarify the extent and nature of *MYB5a* functional divergence in *M. l. variegatus*.

The adaptive significance of this evolutionary change is not yet fully understood. Flower color differences in *Mimulus* can have dramatic effects on pollinator behavior (Schemske and Bradshaw 1999). However, in wild populations in Chile, both *M. l. luteus* and *M. l. variegatus* are pollinated by the native Chilean bumblebee *Bombus dahlbomii*, which did not exhibit either collective or individual flower color preferences in a mixed-phenotype swarm of *Mimulus* hybrids (Cooley *et al.* 2008). Anthocyanins also protect plants against a variety of environmental stressors including extremes of heat, cold, drought, and UV radiation (Chalker-Scott 1999). The closely related *M. cupreus*, which recently gained petal lobe anthocyanins independently of *M. l. variegatus* (Figure 1A), does show greater drought tolerance than the yellow-flowered *M. l. luteus* (Stanton *et al.* 2016), but this work has not yet been extended to *M. l. variegatus*.

Our RNAi results indicate that *MYB5a* is responsible for anthocyanin pigmentation in two distinct regions of the *M. l. variegatus* corolla: the nectar guide and the petal lobe. The role of *MYB5a/NEGAN* in nectar guide spotting appears to be conserved across much of the genus. Its deployment to the petal lobes is unique: in diverse species including *M. lewisii*, *M. aurantiacus*, *M. cupreus*, and *M. naiandinus* (Cooley and Willis 2009; Cooley *et al.* 2011; Streisfeld *et al.* 2013; Yuan *et al.* 2014), petal lobe anthocyanin pigmentation is controlled by ancient paralogs of *MYB5a/NEGAN*, located at the functionally similar but genetically unlinked genomic region *pla1* (Figure 1).

The R2R3 MYB gene *PELAN*, which is responsible for petal lobe pigmentation in *M. lewisii*, differs fundamentally from *MYB5a/NEGAN* in that it does not appear to function as part of a reaction-diffusion network (Yuan *et al.* 2014). Several observations indicate that *MYB5a* in *M. l. variegatus* is operating via reaction-diffusion, despite the apparently unpatterned nature of its petal lobe anthocyanin phenotype. (1) Crossing *M. l. variegatus* to the conspecific variety *M. l. luteus* reveals a pattern of anthocyanin spots in the petal lobes of the F1 hybrids, rather than a spatially uniform reduction in pigment intensity. (2) Knocking down *MYB5a* in *M. l. variegatus* eliminates the nectar guide spots, indicating that *MYB5a* has retained this ancestral ability which relies on reaction-diffusion (Ding *et al.* 2020). Our transcriptome data show that no other Subgroup 6 R2R3 MYB was significantly down-regulated in the MYB5a RNAi line, confirming that the loss of nectar guide spots is unlikely to be caused by an off-target effect of the RNAi transgene silencing a paralogous MYB gene. (3) The RNAi lines with weaker phenotypes did not show a spatially uniform reduction in pigment intensity, as has been observed for RNAi against the non-autocatalytic R2R3 MYB *PELAN* (Yuan *et al.* 2014). Rather, pigment was completely lost in portions of the petal, and the remaining pigment was sometimes expressed in a spotted pattern (*i.e.*, line Vrnai6; Figure 4).

The genomic region harboring *PELAN* appears to have been used for petal pigmentation across *Mimulus* more often than the genomic region harboring *MYB5a/NEGAN* (Figure 1). More work is required to determine whether the inability of *PELAN* to self-activate is a recently derived trait within *M. lewisii*, or a conserved and evolutionarily ancient feature. It is interesting to note that the orthologous (*pla1*) genomic region in *M. guttatus* has been found to create semi-stochastically distributed red dots of anthocyanin pigment on the calyx (Lowry *et al.* 2012), suggesting that the causal gene in this instance is capable of reaction-diffusion-type patterning. While circumstantial, this discovery suggests that the lack of self-activation in *PELAN* in *M. lewisii* may be the exception rather than the rule for anthocyanin regulatory networks in *Mimulus*.

The repeated gains and losses of anthocyanin pigmentation across *Mimulus* (Grossenbacher and Whittall 2011), combined with the genomic and molecular resources that characterize the genus (Sobel and Streisfeld 2013; Twyford *et al.* 2015; Yuan 2019), create fertile ground for investigating how two major developmental approaches to trait formation—reaction-diffusion and positional specification—are utilized in the evolution of biological diversity. One challenge in studying anthocyanin pigmentation by either mechanism is that the number of interactions in the regulatory network makes it difficult to precisely predict the effects of perturbing any one component. Yet, the ability to make such predictions is central to generating and testing mechanistic hypotheses about how the system functions.

Mathematical modeling provides a potentially powerful solution to this problem. Yet, empirical estimates for many of the necessary parameters—such as the strength of the interactions among the different regulatory proteins—are lacking. Discovering the values of such parameters is essential in order to constrain a wide universe of possible models to a narrower range of biologically plausible ones.

**Figure 7:**
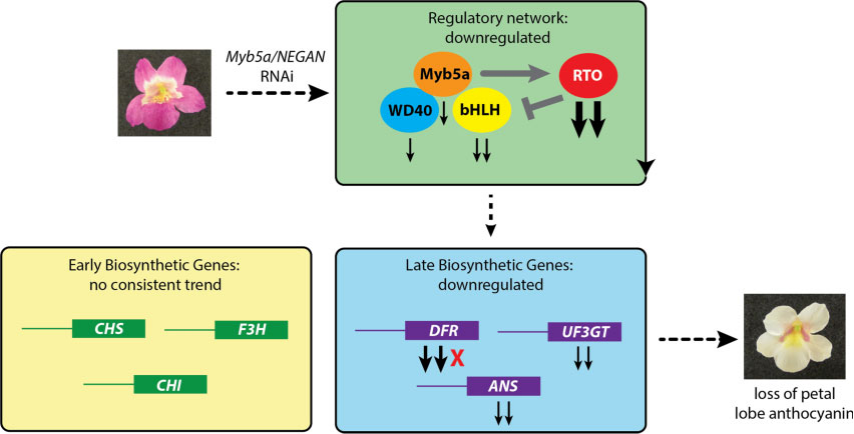
Solid black arrows indicate the direction of gene expression change, following RNAi knockdown of MYB5a/NEGAN in M. l. variegatus. The number of black arrows corresponds to the number of gene copies identified in the transcriptome. Red X, gene expression near zero in the RNAi line; thin black arrow, approximately 3-fold downregulation in the RNAi line; medium black arrow, approximately 9-fold downregulation; thick black arrow, more than 18-fold downregulation. Grey symbols denote positive and negative regulatory interactions. RTO is an R3 MYB protein that inhibits anthocyanin biosynthesis by sequestering bHLH proteins away from the MBW complex.

One striking result from our transcriptome data was that an RNAi knockdown of *MYB5a/NEGAN* had a much stronger effect on the *RTO* repressor than on *MYB5a* itself (Figure 7). To further explore this result, we used mathematical modeling to identify conditions that could give rise to the observed patterns of gene expression change between wild-type and Vrnai1 lines of *M. l. variegatus*. We found that the experimental observation that a knock-down of *MYB5a/NEGAN* decreases the RTo concentration is consistent with activator-inhibitor systems that serve as minimal models of the MBW regulatory network (see Supplementary File S1). Working with such a minimal model, we found it straightforward to identify parameter values that demonstrate how a knock-down of *MYB5a/NEGAN* can result in a relatively larger fold change of the inhibitor concentration. Additionally, by changing parameters, we are able to demonstrate a wide variety of theoretical behaviors consistent with previous experimental results (Ding *et al.* 2020). According to this model, a knock-down of activator will always cause a decrease in inhibitor concentration; the relative size of the inhibitor change will vary depending on the parameter values.

Overall, our transcriptome analyses and models highlight the inter-relatedness of expression patterns across the network of anthocyanin regulatory and biosynthetic genes (Figure 7). These results illustrate how the network can respond dynamically to expression changes of a single network component, creating an avenue for a relatively modest expression change—such as that caused by a single *cis*-regulatory mutation—to have a major effect on the transcriptome as well as on the ultimate phenotype.
